# Zinc Complexation Overcomes the Context-Dependent Metabolic Effects of Curcumin in TNBC: Molecular Insights from TLR4/MD-2 Targeting

**DOI:** 10.3390/cimb48060603

**Published:** 2026-06-06

**Authors:** Giorgia Francesca Saraceno, Gessica Bonavota, Emilia Furia, Erika Cione, Paola Tucci

**Affiliations:** 1 Department of Pharmacy, Health and Nutritional Sciences, University of Calabria, 87036 Rende, Italy; giorgiafrancesca.saraceno@unical.it (G.F.S.); gessicabonavota@gmail.com (G.B.); 2 Department of Chemistry and Chemical Technologies, University of Calabria, 87036 Rende, Italy; emilia.furia@unical.it

**Keywords:** breast cancer, hyperglycemia, natural compound, anti-inflammatory

## Abstract

A critical yet frequently overlooked factor is the tumor’s metabolic profile. Diabetes and chronic moderate hyperglycemia are known risk factors for triple-negative breast cancer (TNBC) that do not respond to hormonal therapy. So, identifying novel therapeutic targets and developing more effective treatments is needed. One of the key pathways involved in the aggressive nature of TNBC is the Toll-like receptor 4 (TLR4) signaling cascade. To this end, curcumin (CUR) has shown effects consistent with modulating inflammatory stress by inhibiting TLR4/MD-2. This study evaluated CUR at concentrations observed in the bloodstream (0.025–25 ng/mL) in MDA-MB-231 TNBC cells under different glucose conditions (normal, moderate, and severe hyperglycemia) and inflammatory states (LPS-induced), using cell viability assays and molecular docking. A zinc complex (Zn–CUR) was also used. Results were validated through cell viability assays. Under severe hyperglycemia, CUR unexpectedly increased cell viability in a dose-dependent manner, while Zn–CUR had no activity across all glucose levels. In LPS-induced inflammation, CUR exhibited a biphasic, dose-dependent response, being protective at mid-level doses but cytotoxic at higher doses, whereas Zn–CUR showed more consistent effects, consistent with modulation of inflammatory stress. Molecular docking suggests that Zn–CUR binds more stably within the MD-2 hydrophobic pocket than CUR, particularly when bound to LPS, with binding energies of −8.7 and −8.3 kcal/mol, respectively. However, better in silico affinity did not always translate into improved cellular effects. These findings indicate that metabolic context significantly influences CUR’s biological activity and that forming a zinc complex offers a safer, more reliable profile. This positions Zn–CUR as a candidate warranting further investigation for TNBC, particularly in the context of hyperglycemia.

## 1. Introduction

Triple-negative breast cancer (TNBC), characterized by the absence of estrogen receptor (ER), progesterone receptor (PR), and human epidermal growth factor receptor 2 (HER2) amplification, is the most aggressive and resistant subtype of breast cancer [1]. Accounting for approximately 15–20% of all breast cancer cases, TNBC is associated with a disproportionately high rate of early recurrence, metastasis, and death, with limited targeted treatment options [2]. Managing TNBC primarily relies on standard chemotherapy, which often yields initial responses but is frequently followed by resistance and disease progression [3,4]. This stark clinical reality underscores the urgent need to elucidate the complex molecular mechanisms driving TNBC, identify novel therapeutic targets, and develop more effective treatments. One of the key pathways involved in the aggressive nature of TNBC is the Toll-like receptor 4 (TLR4) signaling cascade. TLR4 is a pattern recognition receptor (PRR) that serves as a crucial sentinel of the innate immune system, traditionally recognized for detecting pathogen-associated molecular patterns (PAMPs), such as lipopolysaccharide (LPS) from Gram-negative bacteria [5]. However, growing evidence highlights its essential role in cancer development. In the tumor microenvironment, TLR4 can be activated by endogenous ligands, known as damage-associated molecular patterns (DAMPs), released by necrotic or stressed cells [6]. Upon activation, primarily via the TLR4/MD-2 complex, TLR4 triggers a downstream signaling cascade that branches into MyD88- and TRIF-dependent pathways, leading to activation of transcription factors such as NF-κB and IRF3 [7]. This results in the production of various pro-inflammatory cytokines, chemokines, and survival factors, including TNF-α, IL-6, and IL-1β [8]. In cancer, this ongoing inflammation is not just a bystander but actively promotes tumor growth. In TNBC models, TLR4 activation has been clearly shown to increase cancer cell proliferation, support survival against apoptotic signals, and, notably, enhance migratory and invasive abilities, thereby promoting metastasis [9,10]. Clinical studies further show that high TLR4 levels in breast cancer tissues are often linked to worse patient outcomes [11]. In the ongoing search for naturally derived compounds with potent anti-inflammatory and anticancer effects, curcumin (CUR), the main curcuminoid from turmeric (Curcuma longa), has gained significant interest. Its long history of use in traditional medicine is now supported by recent scientific research demonstrating its multiple effects. CUR has been reported to directly interact with the MD-2 co-receptor of TLR4, competing with LPS for binding to its hydrophobic pocket and thereby modulating downstream signaling pathways [12]. Moreover, CUR has been shown to target a wide array of molecular targets, including transcription factors, growth factors, inflammatory cytokines, and protein kinases [13,14,15]. Notably, preclinical studies consistently indicate that CUR can inhibit cell growth, trigger apoptosis, and reduce metastasis in various breast cancer cell lines, including MDA-MB-231 [16,17]. Significant research by Zhou et al. demonstrated that CUR suppressed macrophage polarization and the production of TNF-α, IL-6, and IL-12B by blocking the TLR4-mediated signaling pathway [18]. Despite this promising preclinical profile, the clinical use of CUR has been greatly limited by its inherent physicochemical issues, especially its extremely poor water solubility and rapid metabolism, which lead to low bioavailability [19]. To tackle these problems, various formulation strategies have been studied, including the development of nanoparticles, liposomes, and phospholipid complexes [20,21]. Another innovative method involves forming metal complexes with CUR. The formation of a zinc–CUR complex (Zn–CUR) has been proposed to improve CUR stability and potentially its bioactivity. Zinc is an essential trace element involved in many cellular and immune processes, and its complexation with CUR has been shown to alter the compound’s electronic distribution and flexibility, potentially enhancing its interaction with biological targets [22]. Early studies suggest that metal complexes of CUR could have stronger antioxidant and anti-inflammatory effects than CUR alone [23]. However, research on CUR and its metal complexes in cancer, especially in TNBC, remains incomplete and is often conducted under simplified in vitro conditions that do not reflect the complexity of the tumor environment. A critical yet frequently overlooked factor is the metabolic profile of the tumor microenvironment, particularly hyperglycemia. Diabetes and chronic hyperglycemia are known risk factors for several cancers, including breast cancer, and are linked to worse outcomes [24,25,26]. Hyperglycemia can promote tumor growth through various mechanisms, including increased oxidative stress, formation of advanced glycation end products (AGEs), and chronic inflammation [27]. Importantly, high glucose levels have been shown to influence the expression and activity of TLR4, potentially creating a vicious cycle of inflammation and metabolic imbalance that drives cancer aggressiveness [28,29]. Therefore, assessing the effectiveness of any therapeutic agent without considering glucose levels in the culture medium greatly oversimplifies the situation and can lead to misleading assumptions about its true biological activity and therapeutic potential. Based on these considerations, this study aims to evaluate the effects of CUR and its Zn complex under conditions that more accurately mimic the pathophysiological state of TNBC. By combining cellular assays with computational chemistry, this research seeks to provide a comprehensive, mechanistically informed understanding of the potential of Zn–CUR as a new therapeutic agent for TNBC, a disease that heavily depends on the tumor microenvironment’s metabolic and inflammatory conditions.

## 2. Materials and Methods

### 2.1. Cell Culture and Hyperglycemic Conditions

The human epithelial breast carcinoma cell line MDA-MB-231 was obtained from the American Type Culture Collection (ATCC, Manassas, VA, USA). Cells were cultured in Dulbecco’s Modified Eagle Medium (DMEM; Corning, Cellgro, Manassas, VA, USA) supplemented with 10% (*v*/*v*) fetal bovine serum (FBS; Invitrogen, Waltham, MA, USA) and 1% (*v*/*v*) penicillin-streptomycin. Cultures were maintained in a humidified incubator at 37 °C with 5% CO_2_ and passaged at 80–90% confluence using 0.25% trypsin-EDTA. Every four months, cells were authenticated by short tandem repeat analysis using the AmpFLSTR Profiler Plus PCR Amplification Kit (Applied Biosystems, Monza Brianza, Italy) at our Sequencing Core. Morphology, doubling time, and mycoplasma negativity (MycoAlert, Lonza, ThermoFisher Scientific, Milan, Italy) were tested monthly. To model different glycemic states, glucose levels in the culture medium were carefully adjusted. The standard low-glucose DMEM (D6046, containing 1 g/L glucose = 100 mg/dL) was used as the normoglycemic condition. To obtain hyperglycemic conditions, a sterile D-glucose solution (G8270) was dissolved in sterile water, filtered (0.22 µm), and then added to achieve final concentrations of 175 mg/dL (moderate hyperglycemia) and 350 mg/dL (severe hyperglycemia). Osmolality was measured using a vapor pressure osmometer. Values remained within the physiological range (290–340 mOsm/kg) across all conditions: 100 mg/dL glucose (298 ± 4 mOsm/kg), 175 mg/dL glucose (305 ± 3 mOsm/kg), and 350 mg/dL glucose (315 ± 5 mOsm/kg). To maintain this range, mannitol (final concentration 20 mM) was added to the 100 mg/dL and 175 mg/dL conditions to match the osmolality of the 350 mg/dL glucose condition, ensuring that observed effects are attributable to glucose rather than osmotic stress. To maintain environmental stability, the pH of all culture media was regularly checked and adjusted as needed using a calibrated pH meter with a KCl reference electrode. Media were replaced every 48 h to maintain steady nutrient levels and prevent the buildup of metabolic waste. All chemicals were from Sigma/Merck (Darmstadt, Germany).

### 2.2. Synthesis and Characterization of Zinc–Curcumin Complex

The zinc–curcumin complex (Zn–CUR) was prepared by mixing ZnCl_2_ with CUR in a 1:2 molar ratio under controlled pH conditions using potentiometric titration. The complex precipitated as a reddish-orange solid from aqueous ethanolic solution and was collected by centrifugation and washed with cold ethanol. The stoichiometry and coordination mode were confirmed by potentiometric titration and by analysis of experimental data using both graphical and numerical approaches. Plotting the average number of protons lost per ligand as a function of pH indicated that this value tends toward two, and numerical treatment of the data was consistent with a 1:2 metal:ligand stoichiometry. Complex formation was further confirmed by UV-Vis spectroscopy, which showed significant spectral shifts compared to the free CUR ligand (Figure S1). The proposed structure of the Zn–CUR complex (Figure S1) is consistent with previously reported models for bis(Curcuminato)metal(II) complexes [30,31,32,33].

### 2.3. Cell Treatments

Curcumin (CUR) and its zinc–curcumin complex (Zn–CUR) were used to examine their biological effects in the triple-negative breast cancer (TNBC) cell line MDA-MB-231. Stock solutions of each compound were prepared in dimethyl sulfoxide (DMSO) and then diluted in cell culture medium to reach the final working concentrations. Cells were treated with concentrations of 0.025, 0.25, 2.5, and 25 ng/mL for 24 h. A vehicle control, consisting of culture medium with an equal volume of DMSO (≤0.1%), was included in all experiments to account for any potential non-specific solvent effects. All chemicals were from Sigma/Merck (Darmstadt, Germany).

### 2.4. Cell Viability Assay and TLR4 Stimulation

Cell viability was evaluated using the 3-(4,5-dimethylthiazol-2-yl)-2,5-diphenyltetrazolium bromide (MTT) assay, which assesses mitochondrial succinate dehydrogenase activity. Briefly, MDA-MB-231 cells were plated at 4.5 × 10^3^ cells per well in 100 µL of medium in 96-well plates and incubated overnight to allow adherence. For viability testing under different glycemic conditions, cells were treated with specific concentrations of CUR or Zn–CUR for 24 h. To investigate TLR4 pathway involvement, cells were pre- and post-treated with CUR or Zn–CUR in the presence of ultrapure *E. coli* lipopolysaccharide (LPS), a standard TLR4 agonist, at concentrations of 100 ng/mL and 200 ng/mL. After 24 h, 10 µL of MTT solution (5 mg/mL in PBS) was added to each well, and the plates were incubated for an additional 4 h at 37 °C. Formazan crystals were dissolved by adding 100 µL of DMSO to each well under basic conditions [30]. Absorbance was measured at 545 nm using a microplate reader. Cell viability was expressed as a percentage relative to the vehicle control (DMSO-treated cells), which was set to 100%. All chemicals were from Sigma/Merck (Darmstadt, Germany).

### 2.5. Molecular Docking Simulations

Ligands, CUR and Zn–CUR, were built in ChemDraw (version 16 Pro) and energy-minimized using OpenBabel (version 3.2.0). Docking simulations were carried out using AutoDock Vina (version 1.1.2). Computational molecular docking was performed on the murine TLR4–MD-2 complex, focusing on the MD-2 hydrophobic pocket. The docking grid was centered at x = 2.18, y = 30.95, z = 10.82, with a grid box size of 23.59 × 19.96 × 27.67 Å. The grid was defined based on the position of the co-crystallized lipid IVa ligand, which corresponds to the canonical LPS-binding site within MD-2. Identical grid parameters were used for all simulations to ensure consistency and comparability between CUR and Zn–CUR. Docking simulations were performed both in the presence and absence of the co-crystallized lipid IVa ligand in order to account for ligand-bound and apo conformations of the TLR4–MD-2 complex. The configuration with the best (lowest) binding affinity score from each docking run was selected for further analysis and visualization in UCSF Chimera.

### 2.6. Statistical Analysis

All experiments were performed with at least three independent replicates, and data are shown as the mean ± standard deviation (SD). Statistical analysis and graph creation were carried out using GraphPad Prism 8 for Windows (GraphPad Software, San Diego, CA, USA). Differences among multiple treatment groups were evaluated using one-way or two-way analysis of variance (ANOVA), followed by Dunnett’s post hoc test for multiple comparisons against the vehicle control group. A *p*-value less than 0.05 was considered statistically significant.

## 3. Results

### 3.1. Effects of CUR and Zn–CUR on TNBC Cell Viability Under Metabolic Stress

**Figure 1:**
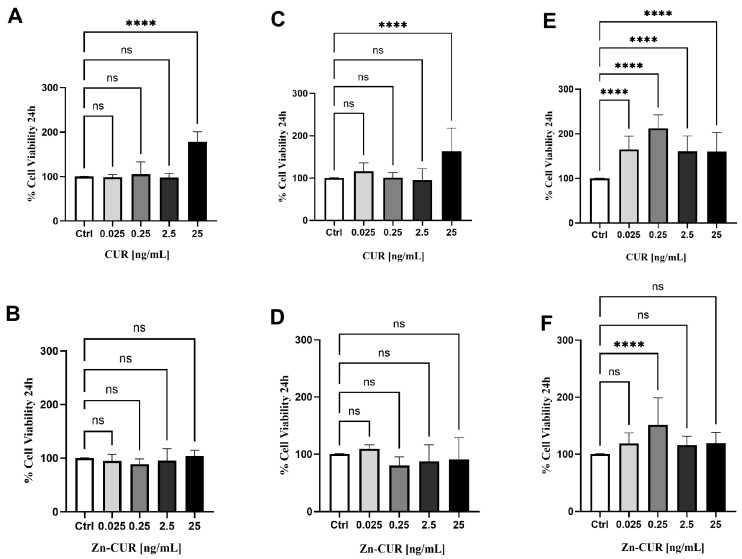
Effects of curcumin (CUR) and zinc–curcumin (Zn–CUR) on cell viability after 24 h under different glucose levels: (**A**) CUR in normal glucose conditions (100 mg/dL); (**B**) Zn–CUR in normal glucose conditions; (**C**) CUR in moderate hyperglycemia (175 mg/dL); (**D**) Zn–CUR in moderate hyperglycemia; (**E**) CUR in high glucose conditions (350 mg/dL); (**F**) Zn–CUR in high glucose conditions. Data are shown as mean ± SD; statistical significance was determined by one-way ANOVA followed by Dunnett’s multiple-comparison test vs. control (Ctrl). Significance levels: ns, not significant; **** *p* < 0.0001.

The effects of CUR and its zinc complex (Zn–CUR) on the viability of MDA-MB-231 cells were assessed under different metabolic conditions: normoglycemia, moderate hyperglycemia, and severe hyperglycemia, using concentrations from 0.025 to 25 ng/mL. Under normoglycemic conditions, CUR significantly increased cell viability only at the highest concentration, 25 ng/mL (Figure 1A), while Zn–CUR showed no effect at any tested dose (Figure 1B). Similarly, under moderate hyperglycemia, CUR induced a significant increase in viability only at 25 ng/mL (Figure 1C), with Zn–CUR remaining biologically inactive across all concentrations (Figure 1D). This overall lack of effect at physiological or moderately elevated glucose levels indicates that neither compound causes overt cytotoxicity nor promotes proliferation under these metabolic conditions. However, under severe hyperglycemia, a notable change was observed. CUR caused a significant, dose-dependent increase in cell viability at all tested concentrations (*p* < 0.001 vs. control), suggesting a potential pro-survival or metabolic adaptation response (Figure 1E). Conversely, Zn–CUR showed minimal biological activity under the same conditions, with only one effect at 0.25 ng/mL (Figure 1F). These results reveal a clear trend: CUR’s ability to promote proliferation rises with higher glucose levels, especially under severe hyperglycemic stress. This context-dependent effect suggests that CUR may activate cellular survival pathways, such as AMPK and Nrf2 signaling, that become more active in a high-glucose environment. In contrast, the consistent inactivity of Zn–CUR across all metabolic states underscores a key difference: zinc complexation appears to neutralize CUR’s ability to stimulate proliferation. This positions Zn–CUR as a promising profile that warrants further investigation as a safer therapeutic candidate, free of unintended growth-promoting effects in tissues under metabolic stress, such as those found in diabetes or tumor environments.

### 3.2. Modulation of LPS-Induced Inflammatory Stress by CUR and Zn–CUR

**Figure 2:**
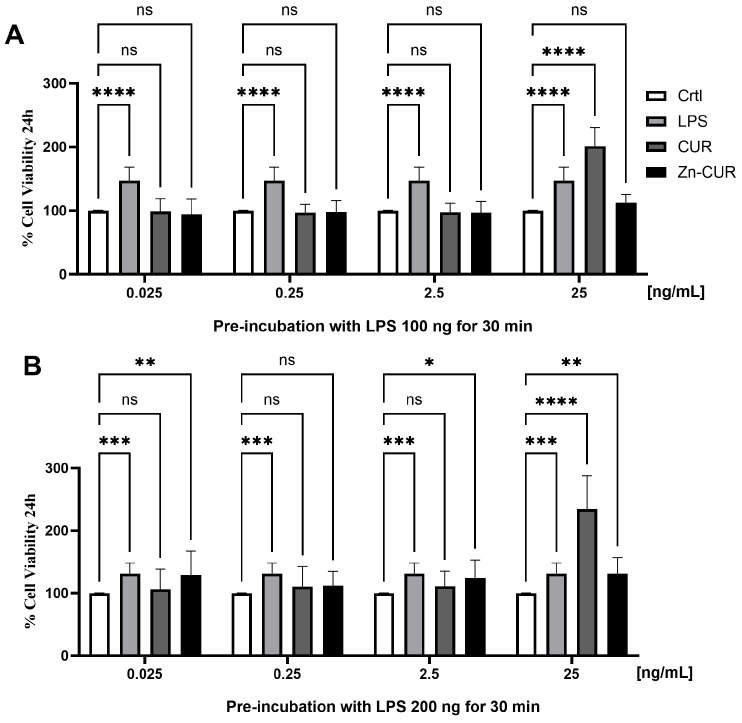
Effects of curcumin (CUR) and zinc–curcumin (Zn–CUR) on cell viability in a post-treatment model. Cells were first stimulated with LPS for 30 min, then treated with CUR or Zn–CUR (0.025, 0.25, 2.5, and 25 ng/mL) and incubated for 24 h. (**A**) Cells treated with 100 ng of LPS. (**B**) Cells treated with 200 ng of LPS. Data are shown as mean ± SD. Statistical analysis was performed using two-way ANOVA followed by Dunnett’s multiple-comparison test. Significance levels: ns = not significant, * *p* < 0.05, ** *p* < 0.01, *** *p* < 0.001, **** *p* < 0.0001.

To assess the modulation of the inflammatory effects of CUR and Zn–CUR, two different experimental protocols were used: a post-treatment model and a pre-treatment model. In the post-treatment model, cells were first stimulated with LPS (100 ng or 200 ng) for 30 min, then treated with CUR or Zn–CUR for 24 h. Initial tests confirmed that LPS activates the TLR4 pathway, a known contributor to oncogenic traits in breast cancer, causing a dose-dependent inflammatory injury in MDA-MB-231 cells, with 200 ng/mL producing a significantly greater effect than 100 ng/mL (Figure 2A,B). Under these post-treatment conditions, neither compound significantly improved cell viability at lower concentrations (0.025–2.5 ng/mL), with only partial protection observed at the highest dose (25 ng/mL) for both LPS doses. This indicates that once inflammation begins, CUR and Zn–CUR have limited ability to reverse LPS-induced changes in viability, likely due to rapid activation of downstream signaling. However, a detailed dose–response analysis revealed a complex, environment-dependent pharmacology. As shown in Figure 2A, at the lowest dose (0.025 ng) following a 100 ng/mL LPS challenge, both CUR and Zn–CUR significantly reduced cell viability compared to LPS alone, indicating an additive harmful effect under mild inflammatory conditions. At a tenfold higher dose (0.25 ng), both compounds markedly increased cell viability in the presence of 100 ng/mL LPS, indicating a protective and modulating effect on inflammatory stress. This biphasic response persisted at 2.5 ng, which continued to provide significant protection at 100 ng/mL LPS. At the highest dose tested (25 ng), CUR became highly cytotoxic under both 100 and 200 ng/mL LPS conditions (Figure 2A,B). Notably, Zn–CUR remained biologically inactive at this high dose. Under the more intense 200 ng/mL LPS condition, the protective effects observed at lower doses were lost (Figure 2B), highlighting the overwhelming influence of acute inflammation. This set of experiments was conducted in high-glucose conditions.

**Figure 3:**
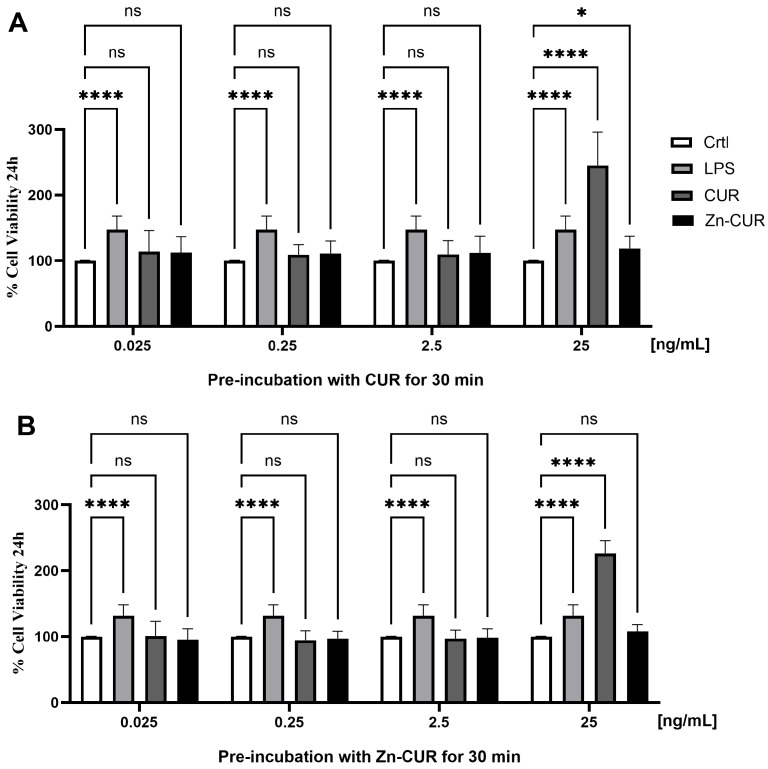
Effects of curcumin (CUR) and zinc–curcumin (Zn–CUR) in a pre-treatment model. Cells were pre-incubated with CUR or Zn–CUR for 30 min and then stimulated with lipopolysaccharide (LPS) for 24 h. Cell viability was measured using the MTT assay. (**A**,**B**) Dose–response effects of CUR and Zn–CUR at 0.025, 0.25, 2.5, and 25 ng/mL. Bars represent the control (Ctrl), LPS-treated, CUR + LPS, and Zn–CUR + LPS conditions. LPS significantly increased cell viability compared with control, while pre-treatment with CUR or Zn–CUR modulated this effect in a dose-dependent manner, with Zn–CUR showing stronger protection at higher concentrations. Data are expressed as mean ± SD. Statistical analysis was performed using two-way ANOVA followed by Dunnett’s multiple-comparison test. Significance levels: ns; not significant, * *p* < 0.05, **** *p* < 0.0001.

These findings demonstrate that CUR exhibits a biphasic effect: it is beneficial at moderate doses against mild inflammation but becomes cytotoxic at high doses (25 ng), regardless of the severity of inflammation. This suggests that CUR’s therapeutic window is narrow and strongly affected by dose and inflammation status. Conversely, Zn–CUR provides a more stable, though somewhat less potent, profile with lower cytotoxic risk at higher concentrations. In the pre-treatment model, cells pre-incubated with CUR or Zn–CUR for 30 min before LPS stimulation showed dose-dependent modulation of the LPS-induced increase in cell viability (Figure 3A,B). LPS alone significantly increased viability compared to control, likely reflecting stress-induced proliferation or cellular activation. Pre-treatment with either compound reduced this effect in a concentration-dependent manner. As shown in Figure 3B, at 0.25 ng and 2.5 ng, both compounds significantly improved viability against severe inflammation (200 ng/mL LPS), demonstrating a protective preconditioning effect. At the highest dose (25 ng), CUR pre-treatment was highly effective, significantly increasing cell viability in the presence of both 100 ng/mL and 200 ng/mL LPS (Figure 3A,B), whereas Zn–CUR pre-treatment at this dose had no effect. Notably, Zn–CUR exhibited stronger modulation at higher concentrations (2.5 and 25 ng/mL) than CUR alone, as evidenced by a more pronounced reduction in the LPS-induced increase in viability. This enhanced effect likely results from the superior bioavailability and stability of the Zn–CUR complex, which may allow more effective interference with early TLR4 signaling, consistent with CUR’s known role as an antagonist of the TLR4/MD-2 complex.

### 3.3. In Silico Prediction

**Figure 4:**
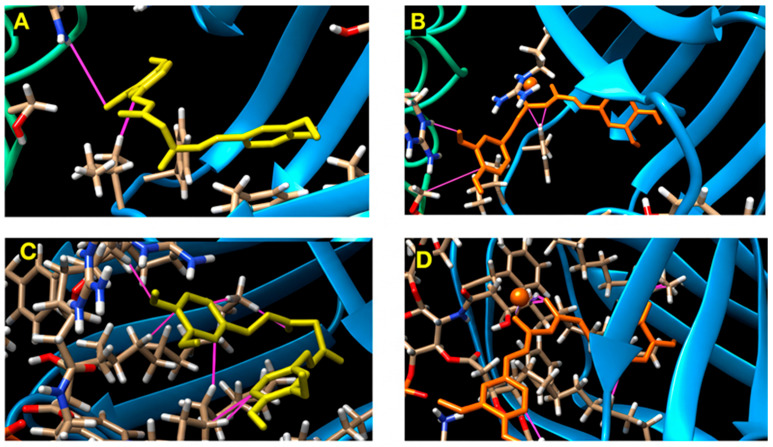
(**A**) Predicted binding pose of CUR (in yellow) in the active site of the TLR4-MD-2 complex in the absence of LPS. The ligand is shown as sticks, highlighting key interactions within the MD-2 hydrophobic pocket. CUR forms several non-covalent interactions with residues lining the binding cavity, indicating moderate affinity and proper orientation for potential biological activity. (**B**) Predicted binding pose of the Zn–CUR complex within the TLR4-MD-2 receptor in the absence of LPS. The zinc coordination with CUR (in orange) is visualized as a metal-ligand complex, shown in stick and sphere forms, respectively. Zinc appears to stabilize the binding conformation, resulting in tighter interactions with MD-2 residues compared to CUR alone. (**C**) Predicted interaction between CUR and the TLR4-MD-2 receptor with Lipid IVa present. CUR is docked into the receptor structure that includes Lipid IVa, representing an LPS-bound state. The pose shows a deeper insertion of the ligand into the MD-2 pocket and a larger interaction surface, supporting the higher binding affinity observed with LPS. (**D**) Top docking pose of the Zn–CUR complex in the LPS-bound TLR4-MD-2 receptor. The figure shows how the Zn–CUR complex fits into the MD-2 pocket while coexisting with Lipid IVa. The conformation demonstrates improved spatial fit and an interaction network that match the strongest binding energy among all tested conditions.

Computational molecular docking was performed on the murine TLR4-MD-2 complex (PDB: 3VQ1) to clarify the biological differences observed between CUR and Zn–CUR, focusing on the MD-2 hydrophobic pocket, a key region involved in LPS (by lipid IVa) binding. The docking grid was centered on the MD-2 hydrophobic pocket at x = 2.18, y = 30.95, z = 10.82, corresponding to the canonical LPS binding site. In the absence of lipid IVa (apo-receptor scenario), CUR was predicted to bind within the hydrophobic pocket of MD-2 with moderate affinity, forming non-covalent interactions that position it for biological activity (Figure 4A), with a binding energy of −7.5 kcal/mol. The Zn–CUR complex showed a more stable binding conformation, with zinc coordination (depicted as spheres) anchoring the ligand in the MD-2 pocket and enabling tighter interactions with the surrounding residues (Figure 4B), as reflected in a slightly more favorable binding energy of −7.8 kcal/mol. This indicates that the zinc ion likely stabilizes the complex. Notably, the presence of the LPS analog lipid IVa increased binding affinity for both ligands, with CUR reaching −8.3 kcal/mol and Zn–CUR achieving −8.7 kcal/mol (Table 1).

This indicates that the zinc ion likely stabilizes the complex, possibly by forming additional coordination bonds or reducing ligand conformational entropy, as evidenced by Zn–CUR’s marginally lower number of active torsions. Overall, the predicted binding poses provide clear structural insights: CUR alone fits within the MD-2 pocket, forming typical non-covalent interactions, whereas Zn–CUR adopts a stable conformation, with the zinc atom likely involved in additional polar or ionic interactions, which may explain its increased binding strength. In the LPS-bound state, both ligands, especially Zn–CUR, penetrate deeper into the pocket, forming an extensive interaction network consistent with the higher affinities observed. These in silico results support the conclusion that Zn–CUR forms a more stable complex with the TLR4-MD-2 receptor than CUR alone. They also reinforce the mechanistic model, suggesting that both CUR and Zn–CUR act as potential MD-2 binders, as indicated by molecular docking. Zinc coordination provides a distinct advantage in binding affinity and stability, thereby enhancing its ability to interfere with TLR4 signaling before ligand activation. The higher affinity, especially when bound to lipid IVa, provides a molecular basis for Zn–CUR’s biological activity and suggests that it may be a more effective agent for interfering with LPS recognition. However, this stronger binding does not always translate into greater modulation of inflammatory stress across all cell-based assays, highlighting the challenge of translating in silico affinity into biological function, where factors such as cell entry, metabolism, and off-target effects play important roles. This suggests that the lipid IVa-bound form of TLR4-MD-2 offers a more accommodating binding pocket for these molecules, likely because LPS binding induces a conformational change that enhances fit or creates new interaction surfaces. In this lipid IVa-bound state, both ligands exhibited increased interaction surfaces and deeper insertion into the MD-2 pocket, consistent with the higher binding affinities observed. Specifically, CUR adopted a pose indicating improved complementarity (Figure 4C), while Zn–CUR demonstrated the strongest overall binding energy, supported by an expanded interaction network and optimal spatial occupancy within the MD-2 pocket (Figure 4D). Nonetheless, these findings position Zn–CUR as a promising, structurally optimized candidate for targeting the TLR4 inflammatory pathway. It is crucial to note that these docking energies represent predicted affinities and serve to generate a mechanistic hypothesis; they do not measure actual binding kinetics or cellular target engagement.

## 4. Discussion

The present study provides a comprehensive assessment of the therapeutic potential of CUR and its zinc complex (Zn–CUR) in TNBC, combining both cellular functional assays under metabolically relevant conditions and molecular docking analyses of TLR4/MD-2 complex interactions. Our findings emphasize a complex, context-dependent pharmacology of these compounds with significant implications for their potential clinical applications. A notable finding of this study is that CUR’s effects on TNBC cell viability are significantly influenced by ambient glucose levels. Under normal and moderate hyperglycemic conditions, CUR showed no cytotoxic effects; however, under severe hyperglycemic stress, CUR unexpectedly increased cell viability in a dose-dependent manner. This observation aligns with recent studies demonstrating that CUR can counteract high-glucose-induced chemoresistance in hepatic carcinoma cells, where hyperglycemia promotes cancer cell survival through metabolic reprogramming [31]. The observed increase in CUR cell viability under severe hyperglycemia may result from activation of the AMPK and Nrf2 signaling pathways, which are typically upregulated during metabolic stress and can improve cellular adaptation and survival [32]. Importantly, this finding shows that CUR’s biological activity depends on the dose and is also heavily influenced by the metabolic microenvironment, an aspect often ignored in standard in vitro studies that use typical glucose levels. In stark contrast to CUR, Zn–CUR exhibited minimal biological activity across all tested metabolic conditions, including severe hyperglycemia. This observation supports growing evidence that Zn–CUR complexes are more stable, soluble, and bioavailable than CUR alone [33]. Recent research shows that Zn–CUR conjugates have better pharmacokinetics, with roughly three times higher cellular uptake than CUR in intestinal epithelial cells and significantly improved systemic bioavailability in animal models [34]. The persistent inactivity of Zn–CUR in our viability tests under hyperglycemic conditions suggests that zinc complexation effectively neutralizes the increase in CUR cell viability, likely by stabilizing the molecule and preventing activation of survival signaling pathways normally triggered by the parent compound. This indicates that Zn–CUR might offer a more favorable profile, lacking the unintended growth-promoting effects in metabolically stressed tissues such as those found in diabetic or hyperglycemic tumor microenvironments. Our investigation of CUR and Zn–CUR in LPS-induced inflammatory models revealed a complex, biphasic dose–response relationship typical of hormetic compounds. At low to moderate doses (0.25–2.5 ng/mL), both compounds showed protective modulation of inflammatory stress effects in the post-treatment model, while at higher concentrations (25 ng/mL), CUR demonstrated significant cytotoxicity. This biphasic pattern is well documented in the CUR literature: a comprehensive review by Moghaddam et al. confirms that CUR consistently exhibits hormetic properties, being stimulatory at low doses and inhibitory at high doses, with low doses often producing stronger effects than higher doses [35]. The differing effects observed between pre-treatment and post-treatment models underscore the importance of timing when using these compounds. CUR and Zn–CUR were more effective when administered before LPS challenge, supporting their role as preventive agents rather than treatments for established inflammation. This aligns with the known mechanism of CUR as an agent that may interfere with LPS recognition by MD-2, with pre-incubation effectively blocking receptor activation [12,36]. The computational docking analyses offered essential structural insights that support and clarify the functional observations. Our results confirm that both CUR and Zn–CUR bind within the hydrophobic pocket of MD-2, the LPS-binding component of the TLR4 receptor complex. This finding aligns with earlier work by Gradisar et al., who demonstrated that CUR binds to MD-2 with submicromolar affinity and that this binding site overlaps with the LPS binding site, thereby inhibiting MyD88-dependent and -independent signaling pathways [12]. Docking simulations were performed both in the presence and absence of lipid IVa to reproduce the receptor’s ligand-bound and apo conformations. This approach enables evaluation of ligand binding across different structural states of the TLR4/MD-2 complex and provides insight into interactions with both the canonical LPS-binding site and the unoccupied pocket. While our docking simulations suggest a plausible molecular basis for the observed cellular effects, direct biophysical confirmation of CUR/MD-2 and Zn–CUR/MD-2 binding is required before concluding that the pathway is inhibited. Importantly, our data show that Zn–CUR has a more favorable predicted binding energy compared to CUR alone, especially in the presence of the LPS analog lipid IVa (Zn–CUR: −8.7 kcal/mol vs. CUR: −8.3 kcal/mol). Zinc coordination appears to stabilize the ligand within the MD-2 pocket, thereby reducing conformational entropy and enabling tighter interactions with nearby residues. This result is consistent with the known ability of phenolic 1,3-diketones, the structural motif of CUR, to coordinate divalent metal ions, thereby improving binding to the MD-2 hydrophobic pocket and influencing LPS-mediated TLR4 signaling [36]. The more extensive insertion and expanded interaction network seen for Zn–CUR in the LPS-bound state provide a structural explanation for its enhanced stability and potentially longer-lasting receptor antagonism. A notable observation is that, despite Zn–CUR’s superior in silico binding affinity, its effects in cellular assays were not consistently more potent than those of CUR. This difference highlights the key distinction between molecular target engagement and overall cellular biological activity. Factors such as cellular uptake, intracellular metabolism, and off-target effects, all influenced by zinc complexation, can significantly affect the final biological outcome. Although Zn–CUR demonstrates improved stability and bioavailability, its changed physicochemical properties might also influence its interactions with other cellular targets, potentially reducing some of the diverse effects observed with the parent compound. This underscores the importance of combining computational predictions with cell-based functional assays under physiologically relevant conditions. The findings of this study have several important implications for the potential clinical use of CUR and Zn–CUR in TNBC. First, the fact that CUR can promote cancer cell growth under severe hyperglycemic conditions raises concerns about its safety for diabetic or hyperglycemic cancer patients, a group that makes up a significant portion of TNBC patients. This is especially relevant given the established link between diabetes and poorer cancer outcomes [31]. Second, the superior safety profile of Zn–CUR across all tested metabolic conditions, combined with its improved MD-2 binding affinity and modulation of inflammatory stress responses, makes this complex a more promising candidate for further development. Zn–CUR’s ability to modulate LPS-induced inflammatory responses without promoting proliferation under hyperglycemic stress suggests it may offer a better therapeutic index than CUR alone. Third, the evidence that both compounds are more effective in preventive pre-treatment than in reversing established inflammation supports their potential use in chemoprevention or for patients with early-stage disease, rather than as a monotherapy for advanced, highly inflammatory tumors. While this study provides important mechanistic insights, some limitations should be recognized. The in vitro experiments, although they allow precise control of metabolic conditions, do not fully capture the complexity of the tumor microenvironment, which includes stromal cells, immune infiltrates, and fluctuating nutrient levels. Future research should incorporate in vivo TNBC models, especially under diabetic or hyperglycemic conditions, to verify the differential effects of CUR and Zn–CUR on tumor growth and metastasis. Additionally, the biphasic dose–response relationships observed highlight the importance of careful dose optimization in future clinical trials, as both too low and too high doses could lead to suboptimal or even harmful outcomes.

## 5. Conclusions

In conclusion, this study demonstrates that the biological effects of CUR in TNBC cells largely depend on metabolic context and dosage, with severe hyperglycemia transforming CUR from an inactive compound into a pro-survival agent. In contrast to CUR, Zn–CUR exhibited limited variation in its biological effects across different glucose conditions, resulting in reduced responsiveness to metabolic changes.

Molecular docking suggests that both CUR and Zn–CUR could interact with the MD-2 hydrophobic pocket, potentially interfering with LPS recognition. However, direct target engagement remains to be demonstrated experimentally. Moreover, it is important to acknowledge that the MTT assay alone cannot elucidate specific mechanisms; accordingly, future studies will integrate complementary assays, including EdU for proliferation, Annexin V for apoptosis, and cytokine quantification for inflammation, to provide a more comprehensive mechanistic understanding.

Overall, these findings provide a valid proof of concept that Zn complexation overcomes the context-dependent effect on cell viability of CUR under severe hyperglycemia, supporting further investigation of Zn–CUR for TNBC, particularly in patients with metabolic imbalance. Future studies should systematically compare Zn–CUR with Cu–, Mg–, and Fe–CUR complexes in terms of MD-2 binding affinity, modulation of cellular inflammatory stress under hyperglycemic conditions, and in vivo efficacy in TNBC models. Such comparative analyses will help identify the most promising metal–CUR complex for translation into clinically relevant settings.

## Figures and Tables

**Table 1:** Binding energies of CUR and Zn–CUR in the TLR4/MD-2 complex in the absence and presence of lipid IVa.

Ligand	Condition	Binding Energy (kcal/mol)
CUR	Without LPS	−7.5
Zn–CUR	Without LPS	−7.8
CUR	With LPS	−8.3
Zn–CUR	With LPS	−8.7

## Data Availability

The original contributions presented in this study are included in the article/Supplementary Material. Further inquiries can be directed to the corresponding authors.

## Supplementary Materials

The following supporting information can be downloaded at: https://www.mdpi.com/article/10.3390/cimb48060603/s1.

## Abbreviations

The following abbreviations are used in this manuscript:

AMPK	AMP-Activated Protein Kinase	LPS	Lipopolysaccharide
ANOVA	Analysis of Variance	MD-2	Myeloid Differentiation factor 2
Ctrl	Control	MTT	3-(4,5-dimethylthiazol-2-yl)-2,5-diphenyltetrazolium bromide
CUR	Curcumin	NF-κB	Nuclear Factor kappa B
DAMPs	Damage-Associated Molecular Patterns	Nrf2	Nuclear Factor Erythroid 2–Related Factor 2
DMEM	Dulbecco’s Modified Eagle Medium	PAMPs	Pathogen-Associated Molecular Patterns
DMSO	Dimethyl Sulfoxide	PBS	Phosphate-Buffered Saline
ER	Estrogen Receptor	PR	Progesterone Receptor
FBS	Fetal Bovine Serum	PRR	Pattern Recognition Receptor
HER2	Human Epidermal Growth Factor Receptor 2	SD	Standard Deviation
IL-1β	Interleukin 1 beta	TLR4	Toll-Like Receptor 4
IL-12B	Interleukin 12B	TNBC	Triple-Negative Breast Cancer
IL-6	Interleukin 6	TNF-α	Tumor Necrosis Factor alpha
IRF3	Interferon Regulatory Factor 3	Zn–CUR	Zinc–Curcumin complex
