# Supplementary material for: Zinc Complexation Overcomes the Context-Dependent Metabolic Effects of Curcumin in TNBC: Molecular Insights from TLR4/MD-2 Targeting
Source: Curr Issues Mol Biol. 2026 Jun 6;48(6):603. doi: 10.3390/cimb48060603 (PMC13298496; doi:10.3390/cimb48060603)
Supplement: Supplementary file 1 [file cimb-48-00603-s001.zip › cimb-4254204-supplementary.pdf]

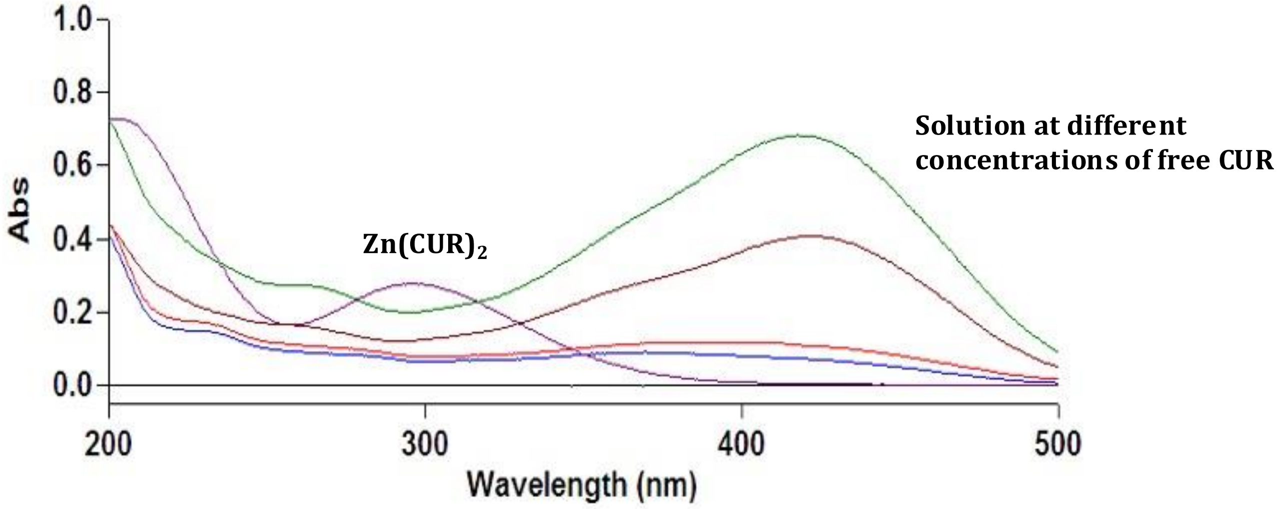

FIGURE S1: The UV-Vis spectra of free CUR and Zn-CUR at different concentrations, demonstrating complex formation through spectral shifts.
